# Chinese expert consensus on standardized treatment for presacral cysts

**DOI:** 10.1093/gastro/goac079

**Published:** 2023-02-21

**Authors:** Gangcheng Wang, Chengli Miao

**Affiliations:** Department of General Surgery, Affiliated Cancer Hospital of Zhengzhou University, Henan Cancer Hospital, Zhengzhou, Henan, P. R. China; Department of Retroperitoneal Tumor Surgery, Peking University International Hospital, Beijing, China

**Keywords:** pelvic cavity, presacral space, presacral cyst, retrorectal tumors, consensus

## Abstract

Presacral cysts are cystic or cyst–solid lesions between the sacrum and rectum, almost involving adjacent pelvic floorstructures including sacrococcygeal fascia, rectum, and anal sphincter. Presacral cysts are usually benign, currently believed to arise from aberrant embryogenesis. Presacral cysts are clinically rare and the true incidence is unknown. Surgical resection remains the major treatment for presacral cysts. Unless the cysts are completely resected, recurrence is unavoidable. Recurrent cysts or hard-to-heal sinuses in the sacrococcyx cause patients extreme pain. However, the current knowledge of presacral cysts is insufficient. They are occasionally confused with other diseases such as ovarian cysts and perianal abscesses. Moreover, lack of the correct surgical concept and skills leads to palliative treatment for complex presacral cysts and serious complications such as impairing the function of the anal sphincter or important blood vessels and nerves. The consensus summarizes the opinions and experiences of multidisciplinary experts in presacral cysts and aims to provide clinicians with a more defined concept of the treatment, standardize the surgical approach, and improve the efficacy of presacral cysts.

## Methodology

### Members of the expert group

The members of the expert group were from two associations (Cancer Prevention and Treatment Expert Committee, Cross-Straits Medicine Exchange Association, and Committee of combined viscerectomy and quality control, Colorectal Cancer Committee of Chinese Medical Doctor Association) and from most provinces, cities, and autonomous regions in China. Major fields included colorectal surgery, gynecological oncology, oncology, radiotherapy, imaging, and pathology, etc.

### Statements

The first consensus draft was written by the experts, and then the relevant problems and rules in the first draft were fully discussed by the experts in the consensus seminar, and the relevant statements were finally formed by experts voting in multiple rounds of meetings.

### Consensus consistency level

(i) 100% voting consensus: all experts reached a consensus and unanimously recommended; (ii) 75%–99% voting consensus: most experts reached consensus and recommended; (iii) 50%–74% voting consensus: most experts reached consensus and recommended, but a few experts disagreed; (iv) <50% voting consensus: not recommended.

## Origin and pathology of presacral cysts

It is a common belief that presacral cysts result from incompletely degenerated primitive embryonic structures during embryonic development, mainly including the tailgut, neural gut, and primitive streak [1–5]. The tailgut is the most distal part of the embryonic gut, which is located at the tail of the cloacal membrane and degenerated completely around the eighth week of embryonic development [6]. The neural gut is a structure connecting the amniotic membrane and yolk sac, which only exists for a few days in the embryonic stage [7]. The primitive streak is mainly composed of totipotent cells, which begin to appear in the third week of the embryo and disappear completely in the fourth week [8, 9].

Presacral cysts are classified into two types: benign and malignant, and most of them are benign, including epidermoid cysts, dermoid cysts, enteric cysts (including tailgut cysts and cystic rectal duplication), neurenteric cysts, teratomas, etc. [10, 11].

### Epidermoid cyst

An epidermoid cyst is a benign single-cystic lesion and contains dry keratinized material on gross macroscopic examination. The cyst wall is only lined with stratified squamous epithelium and without skin appendage structure.

### Dermoid cyst

A dermoid cyst is a benign teratoma of a single germ layer with only ectodermal differentiation, which can be monocystic or polycystic, and a few are solid and filled with thick, cloudy, or sebaceous secretions, and may have hair. The cyst wall is lined with stratified squamous epithelium and there is skin adnexal differentiation, such as pilosebaceous glands or sweat glands.

### Enterogenic cyst

An enterogenic cyst is partially or completely lined with intestinal mucosa and divided into a caudal tailgut cyst and cystic rectal duplication. (i) A tailgut cyst is usually polycystic, with different types of gastrointestinal epithelial cells such as columnar epithelium, squamous epithelium, transitional, or stratified columnar epithelium. The cyst fills with clear, yellowish, or pasty viscous liquid. (ii) Cystic rectal duplication is usually monocystic, lined with the epithelium of the respiratory tract and gastrointestinal tract, and with two layers of muscularis (muscularis mucosa and muscularis propria) and nerve plexus outside.

### Neuroenteric cyst

Compared with the tailgut cyst, the major difference is that a neuroenteric cyst has clear lamina propria and more mature endodermal mucosal differentiation (such as intestinal mucosa and bladder mucosa) [12].

### Teratomas

Teratomas are germ-cell tumors, which are composed of mature tissue of two or three germ layers (ectoderm, mesoderm, and endoderm) [13], and are classified as mature teratomas, immature teratomas, and malignant teratomas in pathology [14]. A mature teratoma is composed of mature tissues and an immature teratoma is composed of immature and mature tissues coexisting in varying proportions. Immature tissues can be derived from three germ layers, mainly neuroectoderm. These immature neuroectoderm structures can occasionally metastasize, mature, or subside spontaneously. The majority of teratomas are benign [15, 16], although the mature teratoma or immature teratoma may be secondary to a malignant teratoma [17, 18].

## Related anatomy of presacral cyst surgery

### Adjacent organs and tissues around the presacral cyst

#### Central presacral cyst

**Figure 1.**
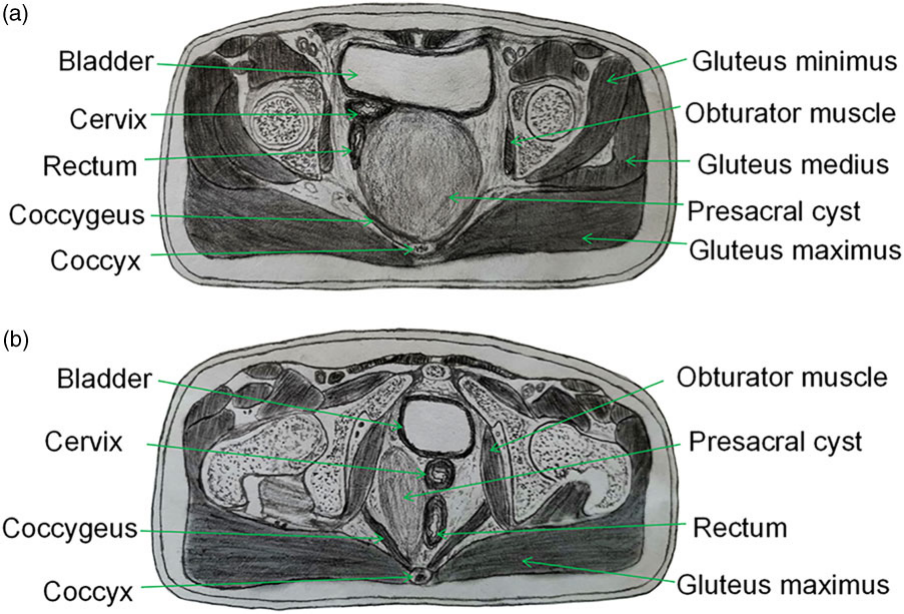
Schematic diagram of presacral cyst types. (A) Central presacral cyst; (B) eccentric presacral cyst.

The main body of a central presacral cyst is located behind the anorectum and in front of the sacrococcyx. The upper pole of the cyst can reach the sacral 2 (S2) level and the lower pole can reach the subcutaneous tissue or even the skin behind the anus. The middle and upper part of the anterior wall of most cysts are densely adhered to the rectal wall or posterior vaginal wall, only a few are membranous adhesions, and the lower part of the anterior wall of the cyst often adheres tightly to the anorectal circular muscle. The posterior wall has a close relationship with the coccygeal fascia and most of them have membranous adhesion with the sacral fascia. If the presacral cyst is complicated with infection or bleeding, the posterior wall will be tightly adhered to the presacral fascia. The walls on both sides of the cyst are adjacent to the gluteus maximus and “U”-shaped levator ani muscle (including coccygeus, iliococcygeus, pubococcygeus, and puborectalis muscles) and parts of the cyst walls reach the ischial tuberosity. Most of the cyst walls adhere to the gluteus maximus and levator ani muscle (Figure 1A).

#### Eccentric presacral cyst

The main body of the eccentric presacral cyst is located on one side of the presacral space and the anterior wall of the cyst is adjacent to the pubis and the muscles associated with the pubis. The adjacent structure of the posterior wall is basically similar to the central presacral cyst. The adjacent structures of the medial wall of the cyst include the vaginal wall (female), prostate (male), rectal wall, anorectal ring muscle group, etc. The lateral wall of the cyst is closely related to the adjacent structures, such as ligaments of ischial tuberosity, gluteus maximus, coccygeus, iliococcygeus, pubococcygeus, and puborectalis muscles (Figure 1B).

However, the coccyx, sacrococcygeal ligament, part of the levator ani, and the external anal sphincter are cut off during the operation, which does not damage the integrity of the anorectal ring muscles, and the internal anal sphincter is not impaired, so the anal function is generally preserved [19–22].

### Associated blood vessels around the presacral cyst

**Figure 2.**
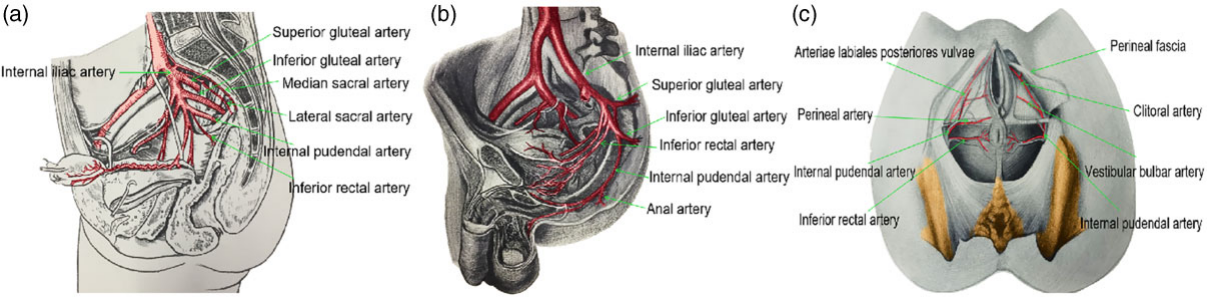
Associated vessels around the presacral cyst. (A) Vessels around the presacral cyst (lateral view); (B) branches of the inferior gluteal artery and internal pudendal artery (lateral view); (C) inferior rectal artery and internal pudendal artery (bottom view).

The associated blood vessels around the presacral cyst mainly include: (i) the lateral sacral artery branches to the sacrococcygeal ligament, accompanying veins and presacral venous plexus; (ii) the terminal branch of the internal iliac artery and vein (inferior gluteal artery and vein), and the internal pudendal artery and vein to the gluteus maximus, levator ani muscle, rectum, and anal canal; (iii) the internal vessels of the mesorectum [23] (Figure 2).

The most common bleeding sites during the operation are sacrococcygeal ligament artery bleeding, internal pudendal artery branch bleeding near the ischial tuberosity, and rectal muscle layer and mesentery bleeding. Most bleeding can be stopped by using sutures or coagulation, although coagulation should be avoided as far as possible when the bowel wall is weak.

### Associated nerves around the presacral cyst

**Figure 3.**
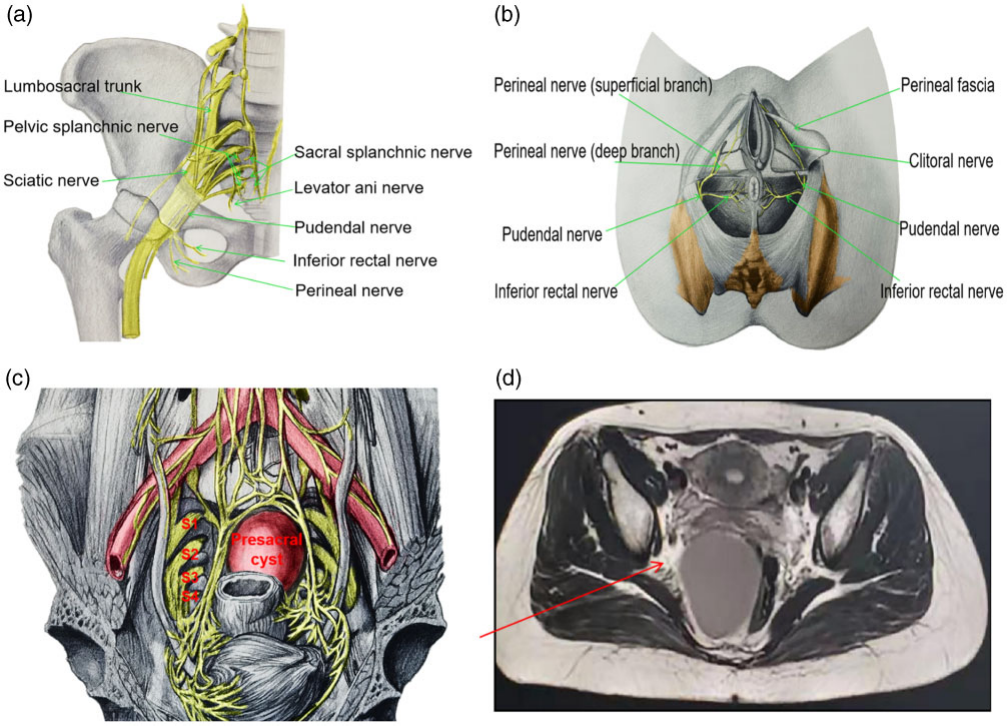
Associated nerves around the presacral cyst. (A) Pelvic and perineal nerves (medial view); (B) perineal nerve branches (inferior view); (C) sciatic nerve and its branches (upper view); (D) magnetic resonance imaging of sciatic nerve (red arrow).

The nerves associated with presacral cyst surgery mainly include the pelvic and perineal nerves [23] (Figure 3). The pelvic nerves mainly include the sacral sympathetic trunk, superior hypogastric plexus, hypogastric nerve, sacral pelvic splanchnic nerve, and pelvic plexus, and the perineal nerves are mainly the inferior rectal nerve and the superficial and deep branches of the perineum from the pudendal nerve.

The nerve injury of presacral cyst resection via an abdominal approach is similar to that of rectal cancer resection via an abdominal approach. For example, the injury of the pelvic visceral nerve may cause dysuria or sexual dysfunction [24, 25]. However, the transperineal approaches involve the perineal nerves, but the inferior rectal nerve and deep branches of the perineum nerves are generally safe from damage, so the transperineal approaches do not affect the patient's defecation, urination, and sexual function.

The sciatic nerve, which is involved in the important function of the lower limbs, originates from the lumbar 4–5 (L4–L5) and sacral 1–3 (S1–S3) nerve roots, passes through the connecting area between S1, S2, S3, and the pelvic wall, passes through the sciatic foramen, and exits the pelvic cavity (Figure 3). Therefore, protection of the sciatic nerve should be highlighted in the dissociation of the upper pole of a presacral cyst.

## Clinical diagnosis of the presacral cyst

### The diagnostic criteria of the presacral cyst

#### Clinical symptoms

Most patients have no specific clinical symptoms and some patients have pelvic organ and nerve-compression symptoms, such as abdominal distension, frequent urination, urgency, difficulty in defecation, abnormal perineum sensation of lower limbs, and habitual abortion, etc.

#### Imaging manifestations

Computed tomography (CT) or magnetic resonance imaging (MRI) shows the cystic or cystic solid lesions behind the rectum and in front of the sacrococcyx, which are closely related to the sacrococcygeal fascia. The cysts show expansile growth and most squeeze the surrounding organs and tissues, and even protrude downward to the subcutaneous tissue of the buttocks and perineum.

Compared with CT, MRI has the advantage of high resolution of soft tissue and can present different signal intensities according to different components in the lesion, which is helpful for identifying types of cysts or other lesions. Meanwhile, MRI can clearly show the location of the cyst and the relationship between the cyst and surrounding important organs and blood vessels through multiparameter and multidirectional imaging. Therefore, MRI is recommended as the first choice for the preoperative diagnosis of presacral cysts.

Endoscopic ultrasonography (EUS) is used to evaluate the origin of the cyst and its relationship with the rectal wall. It can show cystic or cystic solid masses behind the rectum, which closely adhere to the lower rectum but the structure of the rectal wall is complete.

#### Physical examination

Some patients have sunken and wrinkled skin in the posterior midline of the buttock and there is no redness, swelling, heat, or tenderness around the anus. Digital rectal examination or double combination examination can touch the external pressure cystic mass behind the rectum, with poor mobility, no obvious tenderness, and no abnormal rectal mucosa. Abdominal palpation is not easy to perform.

### Differential diagnosis of the presacral cyst

#### Perianal abscess

A perianal abscess usually has infection symptoms, including heat, swelling, pain, and tenesmus, while a presacral cyst does not. During digital rectal examination, a perianal abscess can be touched with a wave motion and obvious tenderness around the rectum, and a presacral cyst only with wave motion. A presacral cyst with infection is easily confused with a perianal abscess, although enhanced MRI can be performed to differentiate them. The MRI of a presacral cyst with infection shows uneven thickening of the cyst wall and obvious enhancement of the signal after enhancement, and a perianal abscess is located in a low position, usually near the anal sphincter. MRI shows typical diffusion-weighted high signal and the abscess wall is enhanced circularly after enhancement.

#### Anal fistula

The internal opening of an anal fistula is located in the rectal cavity and the shape of the anal fistula sinus and the position of the internal opening of the anal fistula can be detected by using sinography or MRI. The sinus of an unhealed presacral cyst is a blind end and angiography or MRI shows that the sinus does not communicate with the rectal cavity.

### Ovarian cyst

Ovarian cysts are located in the pelvic cavity and most of them can be palpated by digital rectal examination and digital vaginal examination. Some tumors are difficult to be pushed due to their large volume, but there is a clear boundary with the sacrococcygeal fascia. Presacral cysts are located in the retroperitoneum of the pelvic floor and are closely related to the sacrococcygeal fascia; the cysts are fixed and cannot be pushed by rectal digital examination. CT or MRI is an important method to differentiate the two diseases.

#### Uterine fibroids

Uterine fibroids are not adjacent to the sacrococcygeal fascia. Active mass can be palpated by digital rectal examination and digital vaginal examination. The imaging findings show solid mass, which is closely related to the uterus. Presacral cysts are located and fixed in the retroperitoneum of the pelvic floor and cannot be pushed by rectal digital examination. The imaging findings are cystic or cystic solid tumors.

#### Presacral neurogenic tumors

Presacral neurogenic tumors are located in the retroperitoneum of the pelvic floor, with a clear boundary with the sacrococcygeal fascia, and most of them have a gap with the rectum. Digital rectal examination and imaging examinations show a solid extra-rectal mass. Presacral cysts are also located in the retroperitoneum of the pelvic floor and are generally closely related to the rectum and sacrococcygeal fascia. Digital rectal examination and imaging examinations suggest a cystic or solid cystic mass with external rectal pressure.

#### Pseudopresacral cyst after rectal cancer surgery

A pseudopresacral cyst is a cystic mass that is not shown on preoperative imaging and appears after surgery. Mostly pseudocysts are formed by mucus secreted by recurrent tumors or residual rectal mucosa, with no adjacent relationship with the sacrococcygeal fascia. However, the primary presacral cysts are mostly congenital, without history of surgery before the first discovery.

#### Rectal stromal tumor

Rectal stromal tumors originate from the submucosal muscle layer and presacral cysts mostly originate from the sacrococcygeal fascia. EUS and MR can effectively distinguish them.

#### Chordoma

When a chordoma grows forward and protrudes into the sacral fossa, the diagnosis of presacral cyst needs to be differentiated from chordoma. The MRI of chordoma mainly shows bone destruction and soft-tissue mass; T2WI and enhanced MRI showed that the low signal fibers in the tumor separate the high signal tumor matrix and tumor cells into multiple lobules, forming a typical “honeycomb sign.” Presacral cysts do not have the above imaging findings.

## Surgical concept of the presacral cyst

### Complete resection of the presacral cyst wall and its closely related sacrococcygeal fascia is strongly recommended [21, 22]

The cyst wall must be removed intact or completely, otherwise it can lead to recurrence of the cyst. The sacrococcygeal fascia may be the origin of the presacral cyst and its remnants can also lead to cyst recurrence.

### Removal of the coccyx is recommended

Because of the close relationship between the coccyx and the sacrococcygeal fascia, resection of the coccyx is recommended to ensure complete removal of the sacrococcygeal fascia.

### Intraoperative use of electrocautery and anhydrous alcohol to disrupt the secretory function of the cyst wall is not recommended

Electrocautery and anhydrous alcohol treatment of the cyst wall cannot destroy the secretory function of the residual cyst wall, which may be a main factor contributing to the recurrence of presacral cysts.

### Use of sclerosing agents to disrupt the secretory function of the cyst wall is not recommended

There is insufficient clinical evidence for the use of sclerosing agents to disrupt the secretory function of the cyst wall. Clinical experience has also demonstrated that presacral cysts treated with sclerosing agents remain secretory and make the removal of presacral cysts more difficult.

### Drainage of presacral cysts is not recommended

Drainage cannot cure the presacral cyst, but instead causes inflammatory edema around the cyst and increases the difficulty of separating the cyst wall from the surrounding organs. Decompression by drainage is not recommended unless the presacral cyst is compressing the rectum or urethra causing difficulty in defecation or urination, or if the patient is physically intolerant to the operation.

### Transrectal or transanal drainage of sac contents is not recommended

When the presacral cyst compresses the rectum and causes defecation disorder or the cyst ruptures and causes surrounding infection requiring emergency puncture and drainage, it is recommended to perform extra-rectal puncture and drainage as far away from the anus as possible and to avoid transrectal puncture and drainage.

### Routine needle biopsy is not recommended

Because presacral cysts are highly tense and have poorly elastic walls, routine needle biopsy tends to increase the risk of infection and sinus-tract formation. For presacral cysts of suspected malignancy (such as patients presenting with intractable sacrococcygeal pain, emission CT suggesting bone destruction, or MRI suggesting invasion of the sacrum and adjacent organs), needle biopsy can be performed to clarify the nature of the cyst.

## Surgical approach for the resection of the presacral cyst (recommended)

### Transperineal approach

#### Longitudinal incision

**Figure 4.**
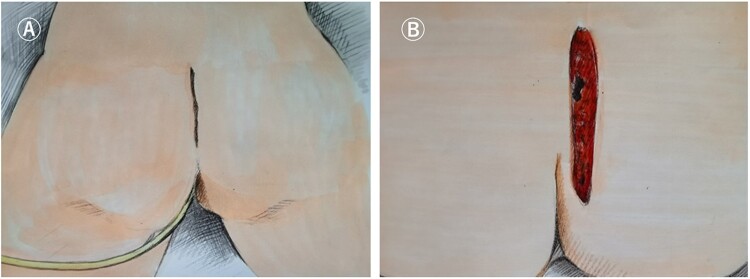
Longitudinal incision (transperineal approach). (A) Schematic diagram of the position of the longitudinal incision; (B) longitudinal incision for presacral cyst resection.

**Figure 5.**
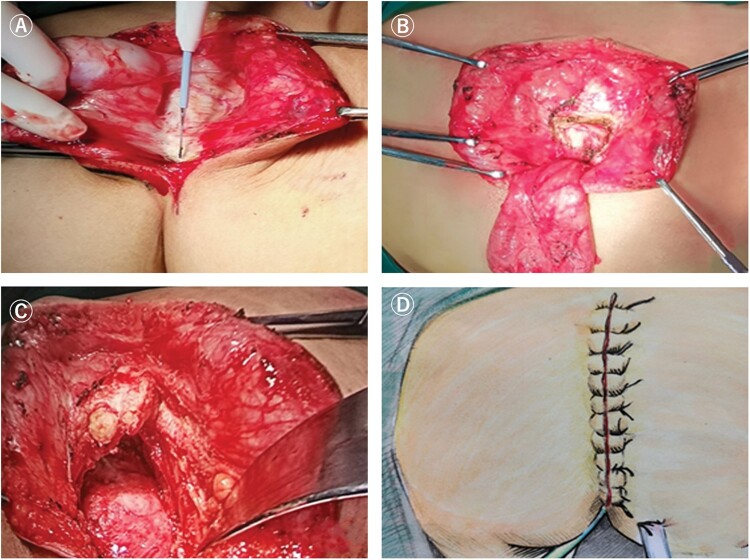
Key points of longitudinal incision for presacral cyst resection. (A) Cut the skin and subcutaneous tissue; (B) expose the sacrococcyx and cut off the coccyx; (C) cut off part of the attachment point of the levator ani muscle on the sacrococcyx; (D) suture the longitudinal incision.

This is recommended for patients with small cysts whose upper pole is lower than the S4 level and can tolerate S4 and S5 vertebral body resection. Patients are placed in the jackknife position and the incision is made along or parallel to the gluteal sulcus [26–28] (Figure 4). During the operation, the attachment of the gluteus maximus and part of the levator ani muscles to the sacrococcyx should be incised, the tip of the coccyx should be removed, and, if necessary, the S4 and S5 vertebrae should be removed. If the cyst is closely related to the rectal wall, the surgeon's fingers are required to enter the rectum to guide the dissection of the cyst wall to protect the rectal wall (Figure 5). The longitudinal incision has the advantages of fast healing and concealed scar, which is suitable for the exposure of eccentric cyst resection, although if the presacral cyst is closely adhered to the presacral fascia, the S4 and S5 vertebral bodies need to be removed to reveal a clear vision for the presacral cyst with a higher position. And if there is a high level of presacral vascular hemorrhage, it is difficult to show blood spots in the jackknife position.

#### Presacral transverse arc incision

**Figure 6.**
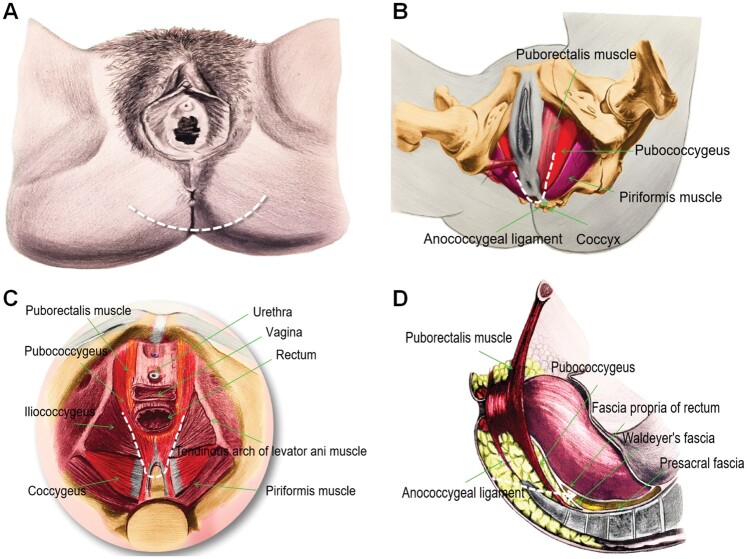
Presacral transverse arc incision (transperineal approach). (A) The location of the arc-shaped transperineal incision anterior to the apex of the coccyx; (B) anatomy of the surgical approach (bottom view); (C) anatomy of the surgical approach (upper view); (D) anatomy of the surgical approach (lateral view) (the white dotted line indicates incision or the surgical approach).

**Figure 7.**
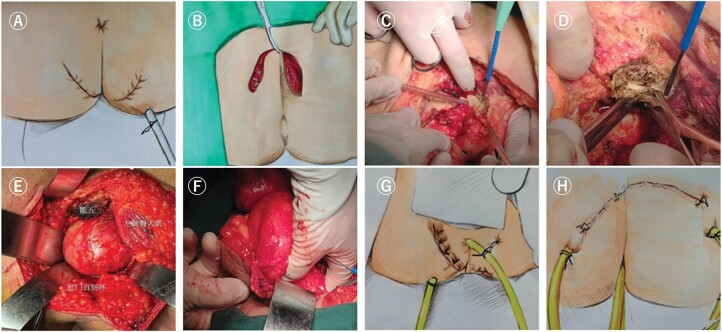
Key points of presacral transverse arc incision for presacral cyst resection. (A) The transverse arc-shaped incision in lithotomy position; (B) the transverse arc incision in prone position; (C) cut off the attachment of the gluteus maximus to the sacrococcyx; (D) cut off the coccyx; (E) expose the presacral cyst; (F) putting the left finger into the rectum to guide the identification of the relationship between the cyst and the rectum; (G) and (H) the placement of the drainage tube and anal decompression tube in the presacral area after operation.

This is recommended for cysts with an upper pole located below S2. For presacral cysts whose upper pole is below S4, the lithotomy position or the jackknife position can be performed, and the jackknife position is better. For presacral cysts with the upper pole above S4, the lithotomy position is recommended. Using the tip of the coccyx as a mark, it is positioned between the tip of the coccyx and the anus, and the left and right are positioned at the inner edge of the ischial tuberosity, connecting three points to form a transverse arc incision [21, 22] (Figure 6). During the operation, the coccyx is cut and the attachment points of the anococcygeal ligament and some gluteus maximus muscles in the sacrum needed to be disconnected. The anorectal ring should be protected and, if necessary, the operator's left forefinger enters the anus to identify the relationship between the anorectal ring or the rectal wall and the presacral cyst. It is recommended to place a presacral drainage tube and an anorectal decompression tube after the specimen is removed (Figure 7).

**Figure 8.**
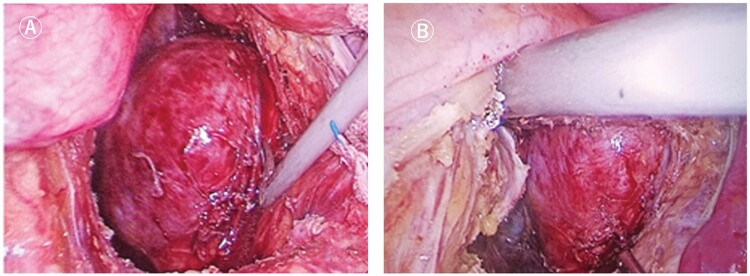
Laparoscopic presacral cyst resection. (A) Exposure of presacral cyst; (B) dissociation of presacral cyst from surrounding tissues.

Compared with the longitudinal incision, the biggest advantage of this incision is to provide enough operation space for cyst separation and presacral hemostasis. However, in the lithotomy position, the tension of the incision is large, which is prone to poor incision healing.

#### *Transperineal pelvic floor intersphincteric incision* [29, 30]

This incision is only suitable for the resection of cysts with very low location and small volume. The patient is placed in the lithotomy position and a “V” incision or radial incision is made behind the anus; the anal canal, internal sphincter, and external sphincter are bluntly separated through the sphincter space, up to the level of the levator ani muscle.

### Transabdominal approach

#### *Laparoscopic presacral cyst resection (Figure 8*)

This is recommended for the initial treatment of presacral cysts with a loose space between the sacrococcygeal and rectal walls, and do not cross the back of the sacrum, and the surgical team is required to have skilled laparoscopic techniques.

The patient's position and trocar position are the same as those for rectal cancer surgery [24, 31]. For some larger presacral cysts, the cyst can be cut open and its contents can be aspirated to expose the operative field. Laparoscopic presacral cyst resection has relatively small trauma and the patient can recover quickly, although there is damage to the hypogastric plexus, which leads to sexual and voiding dysfunctions.

##### Open presacral cyst excision

This is recommended for resecting cysts with a history of open surgery and severe pelvic adhesions, or cysts with a high position that are not suitable for transperineal resection.

### Combined abdominal–perineal approach

This is recommended for presacral cysts with large volume, superior pole higher than S4 level, and inferior pole closely related to the sacrococcygeal region. The lithotomy position with a presacral transverse arc incision or the lithotomy position followed by the jackknife position with a presacral transverse arc incision or longitudinal incision is performed. The presacral cyst is first dissociated to the level of the pelvic floor muscle by laparoscopy or laparotomy, and then the distal end of the cyst is dissociated by the perineal approach. The perineal approach is relatively simple and the incision is relatively small, although the whole operational procedure is relatively complex and the patient’s position needs to change during the operation; there are sexual and voiding dysfunctions caused by the injury of the hypogastric plexus through the abdominal approach.

## Management of perioperative complications of presacral cyst resection

### Management of intraoperative complications

#### Presacral hemorrhage

(i) The presacral bleeding spots are located below the level of S4 and electrocautery coagulation can effectively stop the bleeding. (ii) The presacral bleeding spots are located above the level of S4 and this can be managed by using presacral suture with vascular suture. If not, cotton pads can be used to stop the bleeding by compressing the bleed spots in the presacral residual cavity [32, 33].

#### Rectal rupture

(1) Local repair can be performed and a pedicled greater omentum or transferred muscle flap is used to reinforce the repaired rectal wall [34–37]. (ii) If the local repair is not satisfactory, proximal enterostomy and anal decompression are recommended.

### Management of post-operative complications

#### Delayed healing of incisions at the tip of the coccyx

The incision should be as distant from the anus as possible, which is conducive to reducing incision pollution and improving the blood supply of the flap; the presacral residual cavity and incision are fully drained or rinsed to avoid effusion and infection but if they occur, it is recommended to disassemble the incision for drainage, wipe, or sitz bath.

#### Delayed rectal fistula

The weak rectal wall after cyst separation should be reinforced and preventive enterostomy should be performed when necessary; avoid rectum being corroded by post-operative presacral residual cavity infection, but if it occurs, local resection or repair of the diseased rectum is required and preventive fistula should be performed.

### Anal dysfunction

Anorectal ring muscles should be protected during the operation. If damage occurs, anal-constriction exercises, biofeedback therapy, or magnetic therapy can be performed, and the reconstruction of the anorectal ring muscles can be performed when necessary.

#### Micturition function and sexual dysfunction

The pelvic nerves should be protected as much as possible during operation. If damage occurs, acupuncture, traditional Chinese medicine, and other conservative treatment are recommended.

#### Lower-limb motor or sensory dysfunction

The sciatic nerve and branches should be protected as much as possible during the operation and the cystic fluid can be sucked to better expose the surgical field to protect the nerve for giant presacral cysts. If damage occurs, conservative treatment and functional exercise are recommended, e.g. acupuncture and traditional Chinese medicine.

#### Defecation dysfunction

Diet adjustment is recommended and intestinal function adjustment drugs are used if necessary.

## Follow-up of the presacral cyst

Benign presacral cysts are recommended to be followed up every 6 months within 2 years, and once a year after 2 years. MRI, CT, and ultrasound are recommended to check for cyst recurrence or malignant transformation. If the patient has sudden intractable pain in the sacrococcygeal region after surgery, it may indicate that the cyst has a high possibility of malignant transformation.

Malignant presacral cysts are addressed using supplementary treatment after surgery according to the recommendations of multiple disciplines, such as pathology, orthopedics, anorectal, imaging, radiotherapy, and medical oncology. It is recommended to be followed up every 3 months within 2 years, and every 6 months after 2 years. MRI, CT, and ultrasonography are recommended to be performed for the follow-up.

## Conclusions

**Figure 9.**
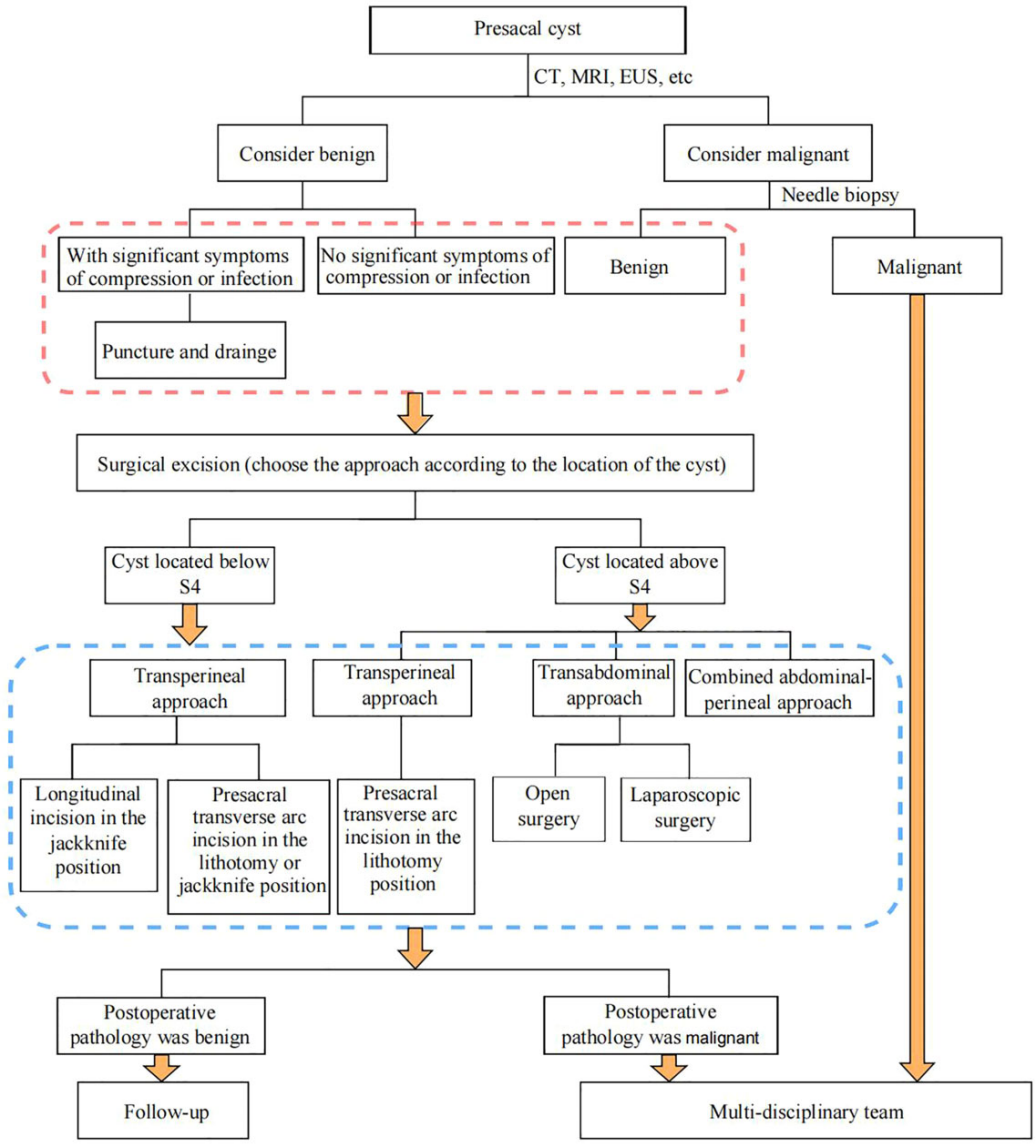
Flow chart of diagnosis and treatment of presacral cyst. CT, computed tomography; MRI, magnetic resonance imaging; EUS, endoscopic ultrasonography.

Presacral cysts, as congenital diseases located in the presacral space, are mostly benign and some have the possibility of malignant transformation. At present, it is believed that their origin is related to abnormal embryonic development, whereas the specific incidence is unknown. Cyst resection is the main cure method and the key point is complete resection of the cyst wall. Several surgical approaches for the resection of presacral cysts have been reported that mainly include transabdominal, transperineal, and combined abdominal and perineal approaches, and each has its own advantages and disadvantages. Most studies on presacral cysts are retrospective, descriptive, or anecdotal, so a prospective, randomized–controlled clinical study needs to be initiated to determine the optimal diagnosis and treatment strategy for presacral cysts, and improve the complete resection rate of presacral cysts, reduce the corresponding injuries and post-operative complications, and finally solve the problem of presacral cyst treatment. A flow chart of the consensus diagnosis of and treatment for presacral cysts is shown in Figure 9.

## List of members of the editorial committee of the “Chinese Expert Consensus on Standardized Diagnosis and Treatment of Presacral Cysts”

Expert group consultant: Jin Gu (Gastrointestinal Cancer Center, Peking University Cancer Hospital), Jianxiong Wu (Department of Hepatobiliary Surgery, Cancer Hospital, Chinese Academy of Medical Sciences), Chenghua Luo (Department of Retroperitoneal Tumor Surgery, Peking University International Hospital).

Head of expert group: Gangcheng Wang (Department of General Surgery, Affiliated Cancer Hospital of Zhengzhou University, Henan Cancer Hospital).

Deputy head of expert group: Lu Yin (Diagnosis and Treatment Center for Difficult Abdominal Surgery, Tenth People's Hospital Affiliated to Tongji University), Xiaojian Wu (Department of Colorectal and Anal Surgery, The Sixth Affiliated Hospital of Sun Yat-sen University), Yudong Wang (Gynecology Department, International Peace Maternal and Child Health Hospital Affiliated to Shanghai Jiaotong University), Xin Wang (Department of General Surgery, Peking University First Hospital), Xiang Feng (Department of Urology, Shanghai Changhai Hospital).

Members of the expert group (in alphabetical order by surname): Songlin An (Department of Peritoneal Oncology Surgery, Beijing Shijitan Hospital), Keyun Bai (Department of Anorectal Surgery, Affiliated Hospital of Shandong University of Traditional Chinese Medicine), Chunqiu Chen (Difficulty Diagnosis and Treatment Center for Abdominal Surgery, Tenth People's Hospital Affiliated to Tongji University), Xiaoxiang Chen (Department of Gynecologic Oncology, Jiangsu Cancer Hospital), Binbin Cui (Department of Colorectal Surgery, Harbin Medical University Cancer Hospital), Yinlu Ding (Department of Gastrointestinal Surgery, The Second Hospital of Shandong University), Wei Fu (Department of Gastrointestinal Surgery, The Affiliated Hospital of Xuzhou Medical University), Yang Fu (Department of Gastrointestinal Surgery, The First Affiliated Hospital of Zhengzhou University), Wenjing Gong (Department of Anorectal Surgery, Zhejiang Provincial People's Hospital), Chunyi Hao (Soft Tissue and Retroperitoneal Neoplasms Center, Peking University Cancer Hospital), Ping Huang (Department of Anorectal Surgery, Sir Run Run Hospital of Nanjing Medical University), Congqing Jiang (Department of Colorectal and Anal Surgery, Zhongnan Hospital of Wuhan University), Haixing Ju (Department of Colorectal Surgery, Zhejiang Cancer Hospital), Yue Kang (Department of Gastrointestinal Surgery, West China Hospital, Sichuan University), Chao Liu (Department of Gastrointestinal Surgery, Sichuan Cancer Hospital), Dianwen Liu (Department of Anorectal Surgery, The Third Affiliated Hospital of Henan University of Traditional Chinese Medicine), Haiyi Liu (Department of Colorectal Surgery, Shanxi Provincial Cancer Hospital), Yingjun Liu (Department of General Surgery, Henan Cancer Hospital), Zheng Liu (Department of Colorectal Surgery, Cancer Hospital, Chinese Academy of Medical Sciences), Changhong Lian (Department of Gastrointestinal Surgery, Heping Hospital Affiliated to Changzhi Medical College), Bin Li (Gynecology Department, Cancer Hospital, Chinese Academy of Medical Sciences), Huichen Li (Anorectal Center of Tianjin People's Hospital), Jun Li (Department of Surgery, Guang'anmen Hospital, China Academy of Chinese Medical Sciences), Ning Li (Department of Gynecology Department, Cancer Hospital, Chinese Academy of Medical Sciences), Taiyuan Li (Department of General Surgery, The First Affiliated Hospital of Nanchang University), Guole Lin (Basic Surgery, Peking Union Medical College Hospital), Yongchao Lu (Traditional Chinese Medicine Department, Shandong Provincial Hospital), Chengli Miao (Department of Retroperitoneal Tumor Surgery, Peking University International Hospital), Wenbo Niu (Department of Surgery, the Fourth Hospital of Hebei Medical University), Debing Shi (Department of Colorectal Surgery, Fudan University Shanghai Cancer Center), Feng Sun (Anorectal Department, The First Affiliated Hospital of Guangzhou University of Traditional Chinese Medicine), Li Sun (Shenzhen Center, Cancer Hospital, Chinese Academy of Medical Sciences), Yi Sun (Anorectal Center, Tianjin People's Hospital), Baochun Wang (Department of Gastrointestinal Surgery, Hainan General Hospital), Guijun Wang (Department of General Surgery, The First Affiliated Hospital of Jinzhou Medical University), Guiying Wang (Department of Gastrointestinal Surgery, The Third Hospital of Hebei Medical University), Hua Wang (Department of Surgery, The Affiliated Hospital of Inner Mongolia Medical University), Lei Wang (Department of Gastrointestinal Surgery, General Hospital of Ningxia Medical University), Meiyun Wang (Department of Medical Imaging, Henan Provincial People's Hospital), Zhigang Wang (Department of Gastrointestinal Surgery, Shanghai Jiaotong University Affiliated Sixth People's Hospital), Jianhong Wu (Department of Gastrointestinal Surgery, Tongji Hospital Affiliated to Tongji Medical College, Huazhong University of Science and Technology), Wei Wu (Department of Geriatric Surgery, Xiangya Hospital, Central South University), Weiqiang Wu (Department of Colorectal and Anal surgery, The 940th Hospital of Joint Logistics Support Force of Chinese People’s Liberation Army [formerly General Hospital of Lanzhou Military Region]), Gang Xiao (General Surgery, Beijing Hospital), Shaomin Yang (Department of Pathology, Peking University Third Hospital), Huiming Yin (Department of General Surgery, Hunan Traditional Chinese Medicine Hospital), Wangjun Yan (Department of Musculoskeletal Surgery, Fudan University Shanghai Cancer Center), Yanling Yang (Department of Hepatobiliary Surgery, Xijing Hospital, Air Force Medical University), Xiaoxia Xu (Nursing Department, Henan Cancer Hospital), Zhiqiang Zhu (Department of Gastrointestinal Surgery, Anhui Provincial Hospital), Xin Zhang (Department of Obstetrics and Gynecology, Emergency General Hospital), Yong Zhang (Department of General Surgery, Zhongshan Hospital Affiliated to Fudan University), Guohua Zhao (Department of General Surgery, Liaoning Cancer Hospital).

Academic Secretary: Yingjun Liu (Department of General Surgery, Henan Cancer Hospital), Guoqiang Zhang (Department of General Surgery, Henan Cancer Hospital), Youcai Wang (Department of General Surgery, Henan Cancer Hospital).

## Conflict of interest

None declared.

## Notes

Cancer Prevention and Treatment Expert Committee, Cross-Straits Medicine Exchange Association.

Committee of combined viscerectomy and quality control, Colorectal Cancer Committee of Chinese Medical Doctor Association.

This consensus is contemporaneously published in *Chinese Journal of Oncology* in Chinese.
